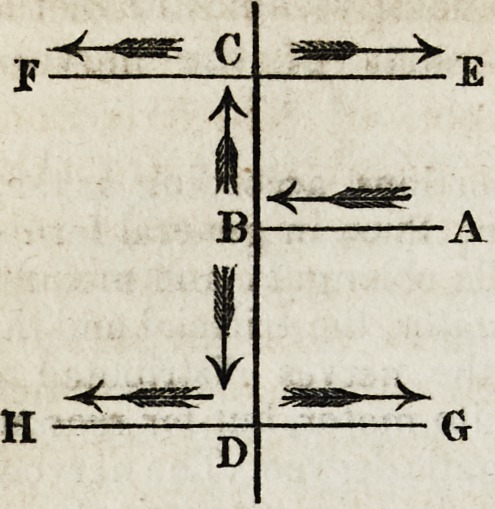# On the True Spinal Marrow, and Its Anatomy, Physiology, Pathology, and Therapeutics

**Published:** 1844-01

**Authors:** 


### Art. X.

1. *New Memoir on the true Spinal Marrow, and its Anatomy, Physiology, Pathology, and Therapeutics.* By Marshall Hall, M.D. F.R.S. L. & E. —*Lond.* 1843. 4to, pp. 94, with Five Plates.

2. *On the Structure, Relations, and Development of the Nervous and Circulatory Systems, and on the Existence of a complete Circulation of the Blood in Vessels, in Myriapoda, and Macrourous Arachnida. First Series.* By George Newport, Esq., President of the Entomological Society of London, Corresponding Member of the Philomathic Society of Paris, &c. 4to, pp. 60, with Five Plates. (*From the Second Part of the Philosophical Transactions for* 1843.)

I. The present memoir, Dr. M. Hall informs us, may be considered as supplementary to his two former memoirs, published in 1837; and as illustrative of his subsequent volume, on the 'Diseases and Derangements of the Nervous System,' published in 1841. To us it seems to contain little that is not embodied in his last-mentioned work, save some criticisms upon his critics; and as we have so recently and fully noticed this, we shall confine ourselves on the present occasion to those questions, on which Dr. Hall throws a new light in the memoir before us. We shall, first, however, in justice to him, quote his *present* statement of his claims, to the general truth of which we fully assent; and if our readers will take the trouble to consult and compare our former articles on the subject, they will find that we can do so without the slightest inconsistency.

"I have reflected with much satisfaction, that, of my various statements, I have scarcely had one to retract, after all the criticism which has been somewhat lavishly bestowed upon them : and that, of the statements of others, scarcely one has either added anything to what had been done, or has proved correct. [We presume that Dr. M. Hall here refers to the *physiological* part of his system; the *anatomical* he has confessedly left to others to work out.] I regard these events as the reward of the care which I had taken to restrain myself within the limits of the most obvious facts, and of the most immediate deductions from those facts. Thus I have restricted the reflex actions to the true spinal marrow. Those who have imagined that a similar reflex function belongs to the cerebral or to the ganglionic system, have, I think, only confused the subject, and added what is really hypothesis or conjecture. I have also confined the part performed by the reflex function within its just limits. Some writers, having extended its influence beyond those limits, have again caused a doubt to be cast over the whole system. In the actions of walking in *man*, I imagine the reflex function to play a very subsidiary part; although there are, doubtless, facts which demonstrate that the contact of the sole of the foot with the ground is not unattended by a certain influence upon the action of certain muscles. It is very different in the *bird* and *fish* tribes, as I shall have occasion to explain hereafter.

"Some writers have taken very unnecessary trouble to prove that the experimental fact of reflex action has long been known to physiologists; as if this had ever been denied, or as if it had not been admitted in the most express terms. (See my First Memoir, § 107.) Others have even descended to show that the term reflex had been employed by former physiologists! (Arnold.) I have never claimed either to have been the first to observe the facts of the reflex action in experiments, or to have been the first to use the term reflex. My real objects have been :—First, to separate the reflex actions from any movements resulting from *sensation* and *volition ;* secondly, to trace these actions to an acknowledged source or principle of action in the animal economy—the *vis nervosa* of Haller—acting according to newly-discovered laws; thirdly, to *limit* these actions to the *true spinal marrow,* with its appropriate incident and reflex nerves, exclusively of the cerebral and ganglionic systems; fourthly, to apply the principle of action involved in those facts to *physiology*—viz. to the physiology of all the acts of *exclusion,* of *ingestion,* of *retention,* and of *expulsion,* in the animal frame; fifthly, to trace this principle of action in its relation to *pathology*—viz. to the pathology of the *entire class of spasmodic diseases ;* sixthly, to show its relation to *therapeutics,* and especially to the action of *certain remedial and certain deleterious physical agents ;* finally, it is to these objects taken together as a *whole* or as a *system,* that I prefer my claims; and I do not pretend that an occasional remark may not have been incidentally made by some previous writer, bearing upon some one or more of them." (pp. 4-5.)

"No better idea of the importance of this discovery can be adduced, than the total obscurity and confusion which are observed to prevail in the best works on the nervous system, written previously to its promulgation. In Legallois we have the utmost confusion in regard to sensation and volition, in their relation to their seat in the nervous system and to the reflex motions; in the work of M. Brachet we have the same confusion in reference to the reflex actions and the functions of the ganglionic system ; and every physiologist remembers, what is so forcibly expressed by Professor Müller, that this latter part of the nervous system was supposed to explain all the sympathies, whence the designation great sympathetic." (pp. 6-7.)

It is but just to the British school of physiology, however, that we should remark, that the erroneous view last adverted to never prevailed in this country among the best-informed physiologists; and that, although the automatic movements were ordinarily confounded with those in which sensation and volition are concerned, they were attributed to the cerebro-spinal system of nerves,—although very far from being properly defined or systematized.

The following is Dr. Hall's general view of the relative amount of the excito-motor power, as possessed by different parts of the nervous system:

"The nervous system may be divided into different portions, according as they are endowed or unendowed with the excito-motor power; thus, in general terms, the cerebrum and cerebellum are *in*-excitor; the medulla oblongata and medulla spinalis are excitor; the nerves of special sense are in-excitor, the trifacial and the analogous spinal nerves are incident excitor nerves; the nerves distributed to muscles are direct excitors; the ganglionic system is excito-motor, but for reasons which will be given hereafter, in a less prompt and energetic degree. The nervous system may therefore be viewed in its cerebral or in-excitor, its true spinal and excitor, and its ganglionic or slowly-excitor portions. The in-excitor portions of the nervous system coincide with the seat of the mental functions. The excitor portions are chiefly dedicated to the acts of ingestion and egestion. The less excitor or ganglionic portions are the nervous agents in secretion, &c. The true limits of the excito-motor power have been accurately determined by M. Flourens, who also ascertained that whilst this property acts in a direct manner in the medulla oblongata, the medulla spinalis, and the muscular nerves, its action is *crossed* from the tubercula quadrigemina or, rather, the peduncles." (pp. 17-18.)

Dr. Hall next endeavours to develop his views respecting the new laws of action of the *vis nervosa*, which he claims to have discovered. On this point, as our readers may recollect, we have on former occasions expressed our inability to discern the novelty of Dr. Hall's doctrines; since it seems to us that the principal change is a verbal one,—namely, his employment of the term *vis nervosa* in a sense in which it had not been previously used. As, however, he considers the right understanding of the laws in question to lie at the foundation of his physiology, we shall place them before our readers, that *they* may benefit by them if *we* cannot.

The *vis nervosa* of Haller is that power, which, travelling along the nervous trunks, excites the muscles to contraction; and, according to Haller himself, as well as later physiologists, this power acts only in the *peripheral* direction,—that is, from the centre to the circumference, the roots to the trunks, the trunks to the branches, the branches to the muscles. "The motor power in the nerves," says Professor Müller, "acts only in the direction of the primitive fibres proceeding to the muscles, or in the direction of the ramification of the nerves, and never in a retrograde direction;" and, "all motor fibres act in an isolated manner, *from* the trunks of the nerves *to* the smallest ramifications."

"As long," says Dr. Hall, "as these views were entertained by physiologists there could be no application of their principle of action, discovered by *experiment*, to actual *physiology;* for, with *one* exception—that of the tone of the muscular fibre—*every* physiological action of the *vis nervosa* or the excito-motor power proceeds along the nerves, in the first instance, in an incident direction *from* their ramifications *towards* their trunk, and thence into the true medulla; *from* and *by* which latter it is *reflected* along other nerves in the direction described by the physiologists whom I have quoted. I have ascertained by a series of *experiments* that the excito-motor power does act in these incident and reflex directions; and I consider the correction of the former error and the establishment of this fact as the first step towards the application of this power to the science of physiology." (pp. 22-3.)

Of the experiments on which Dr. Hall rests his proof of these rather startling positions, we shall cite the *one* which includes all the rest; giving at the same time a rough imitation of the illustrative diagram.

"If a lateral nerve (AB) running from the spinal marrow and truncated at a certain distance from it be stimulated, the anterior and posterior extremities (CE, CF, and DG, DH,) are again moved by the contraction of their muscles. The excito-motor power acting first in an *incident* direction (AB) towards the spinal marrow, then in a *reflex* manner *within* the spinal marrow; (BC and BD, the former being *retrograde*, according to Dr. Hall, the latter *direct;*) and, lastly, in a direction *from* the spinal marrow and *towards* the nervous system, (CD, CF, DG, DH.) If a nerve near the anterior extremities be stimulated, the muscles of these extremities are most moved; if a nerve near the posterior extremities be stimulated, these are most moved in their turn: if a nerve intermediate in its situation be stimulated, the anterior and the posterior extremities may be moved equally.

"It will be observed that these facts are in direct bopposition to the exclusive *law* of action of the excito-motor power propounded y Haller, Prof. Müller, &c. They establish, indeed, a *new law of action* of this power, on which the whole of its physiology depends." (pp. 24-5.)

It appears, then, that Dr. Hall regards the *vis nervosa* or *motor power* of Haller and Müller as *originating* at the point A, and as merely *reflected* by the spinal cord at the point B. If this could be *proved*, a new law of its transmission would certainly be established by Dr. Hall's experiments. But we can find no proof whatever of the *identity* of the influence travelling centripetally along A B, and centrifugally along C E, or D G. It may, or may not, be the same. The plain deduction from the experiment in question, and all others of the same nature, seems to us to be this: that a stimulus applied at A gives rise to *an* influence which travels along the incident trunk A B, until it reaches the spinal cord. The trunk there enters the ganglionic centre of the cord,—the seat, as we have reason to think, of all that is peculiar in its functions; and from this centre proceed the *motor* trunks, which may pass either up or down the cord before making their exit from it to the muscles, which they stimulate to action. We think, in the absence of any proof to the contrary, that we are still entitled to consider, with Haller and Müller, that the *vis nervosa*, or *motor* power, originates in the nervous centres; and that, just as the will or an emotion is the stimulus which occasions its transmission when voluntary or emotional actions are to be produced, so the *excitor* power (to use Dr. Hall's own phraseology) conveyed by the incident nerves, is its stimulus in the case of the reflex actions. It seems to us that we might just as much speak of *contractility* as a property of the motor *nerves*, because their agency throws the muscles into contraction,—as attribute a *motor* power to *incident* nerves, because their agency excites that motor power in the spinal cord. If the spinal cord (and the ganglia corresponding to it in the invertebrated animals) be not the source or centre of the motor power, why should the incident nerves pass into it at all? If *they* convey motor power, why should they not at once go to the muscles on which they are to act? It is surely not enough to say that the spinal cord *reflects* the impulse (as a mirror reflects a ray of light;) for there would be no need of such reflection, if the fibres did not go completely out of their course to enter it. Take, for example, the case of the closure of the lids by the orbicularis, on the application of a stimulus to the tarsal edges, in an animal whose cerebrum has been removed or rendered powerless by a blow. The irritation of the surface excites a change in the extremity of the fifth nerves distributed upon it, which is conveyed along the incident trunk of that nerve to the spinal cord; from the latter proceeds the seventh pair of nerves, which, as it calls muscles into action, we term a motor trunk; and a change in the state of this trunk is made, by which the orbicularis is called into action to close the lid.

Now, have we any authority for stating, that the motor power, which was brought to the orbicularis by the seventh pair, *originated* in the extremities of the fifth, and travelled *towards* the spinal cord at first? Or is it not more conformable to our general ideas on the subject, to regard it as originating in the spinal cord, in virtue of the stimulus or excitation which this organ has received from the impression conveyed to it by the fifth pair? We hope we shall not be suspected of any tendency to undervalue or depreciate Dr. Hall's merits, by urging these views upon our readers. It does not seem to us that there is any important *practical* difference between our notions and his; but we fear that this part of his doctrines may prove a stumbling-block in the way of some, who would otherwise receive his system without difficulty; and that being a theory which *is not* required for the explanation of the facts, it may give an erroneous impression as to that which is. We think it a pity that Dr. Hall does not constantly adhere to the phraseology he elsewhere employs, which is liable to no exception. Thus, in § 152: "I believe that, before the date of my investigations, such an idea or view of *incident nerves*, acting in and through their connexion with the true medulla spinalis and certain *reflex motor nerves*, did not exist in anatomy or physiology." Why will he talk of *incident motor* nerves as having a demonstrated existence?

In the section on the nature of the Excito-motor power, Dr. Hall dwells much upon the necessity of the circulation of arterial blood through the spinal cord, as a condition of its manifestation; but, successfully, as we think, combats the idea of Müller, that the circulation of arterial blood through the medulla oblongata is the *primum mobile* of the respiratory movements. The following observation is of much interest:

"In the very young [warm-blooded] animal, and in the cold-blooded animal, the phenomena of the excito-motor power are far more vividly manifested than in the older and the warm-blooded. In the very young kitten, even when asphyxiated to insensibility, every touch, contact, or slight blow—every jar of the table, any sudden impression of the external air, or that of a few drops of cold water—induces at once energetic reflex movements and acts of inspiration. The nostrils, the tail, the soles of the feet, the general surface, are all extremely susceptible, and in degree in the order in which I have mentioned them. Hence, nature's provision for the first establishment of the first acts of inspiration, and of extra-uterine life. Hence the principle of resuscitation from congenital asphyxia; a subject to which I shall recur in the sequel. *Immediately* after decapitation, or the sudden amputation of a limb, a diminished condition of the reflex excito-motor power is observed:......the animal was placed under the influence of *shock*. In addition to the sources of excito-motor phenomena already noticed, I must now add another: in irritation of any or all of the internal membranes and tissues, serous, mucous, cellular, &c., by means of the scalpel or forceps, in the kitten or the dog, (these were the animals in which I have witnessed these results,) various spasmodic movements are observed. What *physiological* object there may be in these phenomena I do not at present perceive, but it is plain that they present a newly-opened and expansive field of investigation to the pathologist." (pp 29-30.)

The following section, on the 'Appearances of Design,' we shall quote in full, on account of the admirable mode in which the argument is stated. We have ourselves urged the very same considerations, in commenting upon Prof. Volkmann's objections, in a former volume. "The mere adaptation of particular movements to designed ends," we remarked, "cannot be regarded as a reason for supposing that these movements are voluntary, or even dependent upon sensation. The whole organized fabric is made up of a number of such adaptations. The motions of the heart, alimentary canal, &c., are instances of their simplest forms. The motion of the pupil is a case more directly to the purpose, being a reflex action evidently adapted to a particular object,—the conservation of the eye in the state best adapted for the visual function; yet every one knows this to be independent of volition, and pathology shows that it may be performed without sensation." (See vol. VI, p. 213.)

"Every physiological act of the reflex excito-motor power is obviously designed; the act of deglutition, the act of inspiration, the closure of the eyelids, of the larynx, the action of the sphincters;—all is replete with obvious design. How, therefore, the appearance of design in some of the reflex actions observed in experiments should have been imagined, as they have by my venerable friend Prof. Nasse, by Prof. Volkmann, and others, to indicate the presence of a *mental* operation, I cannot comprehend. Thus, if in the decapitated or divided tortoise, we irritate the tail, the posterior extremities are protruded towards the part, so as apparently to remove the cause of irritation; if we pinch the integuments, the extremities are retracted; if we irritate the anus, the limbs are moved so as to be brought into contact with the part. The *design* in all this is obvious; but is it design in the decapitated or divided animal? Certainly not: but of its omniscient Creator! It coincides with what would be design in the animal; otherwise, in such acts of the living and perfect animal, the act of volition and the act of the reflex excito-motor powers would counteract and frustrate each other. We may, I believe, be so far allowed to reason from final causes. And may we not be allowed to say, it is all beautiful, and demonstrative of the wisdom of Him who fashioneth all things after his own will? It is in this manner that the march, the flight,—yes, and the inspiration of birds and of insects, coincide in effecting the combined objects of locomotion and of a most vital function. It is in this manner that we are enabled to contemplate the migration of animals, as effected and continued, however long, like the acts of inspiration, on a principle not susceptible of fatigue. I suspect, indeed, that the migratory traveller is frequently actually visited by nature's sweet restorer, during its aerial transit! In this manner, the ostrich pursued its course, after decapitation by the crescent-headed arrow of the Roman emperor; and the decapitated cock of Kaaw Boerhaave ran on in the direction towards its food, previously impressed by its volition; each successive contact of the foot with the ground exciting the subsequent movement." (pp. 31-2.)

The drawing-up of the limbs of the frog, which takes place after the division of the spinal cord, and which has been referred to by Prof. Volkmann as a *spontaneous* action, denoting sensibility, is thus simply accounted for by Dr. Hall:

"In the first instance, after the division of the spinal marrow, the animal is under the influence of *shock*, and the excito-motor power, with the reflex actions, are suspended for a time. After a short interval, however, this power, with its phenomena, return; the limbs stretched out are then stimulated by its firm pressure against the table, and an excited action, with the retraction of these limbs, occurs." (p. 35.)

In regard to the *distinct anatomy* of the excito-motor system of nerves. Dr. Hall still expresses himself as caring little about the solution of the question. He considers Mr. Grainger's deductions, with respect to the relative offices of the gray and white portions of the cord, as *probable*, but not *proved;* and of the view of Dr. Carpenter and Mr. Newport, respecting the distinct anatomy of this system, in the Articulata, he merely says, " I doubt not that the investigations of these gentlemen are correct; they have, therefore, confirmed what I had long previously done. But it adds nothing, or very little, to the argument, to ascertain that that which is true in one class of animals, proves to be so in another or others." . . . . " It has always appeared to me," says Dr. Hall further on, " that, observing the difference between the cerebrum and the spinal marrow, the olfactory and the trifacial nerves, in regard to their psychical and excito-motor properties, it is very improbable that in *any* part of the nervous system the two functions should coexist in any one individual fibre. The difference of function implies a difference of structure; the difference in physiology implies a corresponding difference in the anatomy. But I am weary of the wordy discussions on this subject; these, therefore, I leave to those who have leisure and taste for them." Now let us analyse this statement, and apply the same line of argument to another case. Dr. Hall has proved, to his own satisfaction, (and, as we freely admit, to ours also,) the existence of a set of nerves *physiologically*—by which we presume he means *functionally*—distinct from those which are concerned in sensation and voluntary movement. But he has not attempted to prove, that these nerves are *anatomically* or *structurally* distinct; and has, in fact, continually expressed himself to the effect that he did not consider such a proof of any consequence. Now if the question were left in this state, its condition would be *precisely the same* as that of the question between the motor and sensory nerves, previous to the discoveries of Sir Charles Bell. Everybody knew THEIR *physiological* distinctness, and some guessed at their *anatomical* distinctness;* but the prevalent opinion, sanctioned by the authority of Haller, was, that the very *same* fibril may be *sensory*, when the change it conveys or communicates is travelling from the periphery to the central organs; or *motor*, when it propagates an influence from the nervous centres to the muscles. In what, then, did Sir C. Bell's merit consist, if there be none in showing that nervous fibres have distinct offices, in virtue of their different connexions with the central and peripheral organs?—in other words, that the fibres which are *physiologically* distinct, are *anatomically* distinct also? In the same manner it might be argued, and *has been* argued, in regard to Dr. Hall's distinct excito-motor system, that although an ingenious hypothesis, it has no real foundation, *because its distinct anatomy cannot be shown;* as there is no *proof*, however *probable* it may seem in Dr. Hall's eyes, that the very same incident fibre may not serve to convey the stimulus for reflex action to the cord, and a sensory impression to the brain; or that the very same motor fibre may not convey to the muscles the influence of the reflex action of the cord, and of the voluntary impulse originating in the brain. This, in fact, is the very

* It appears that *Albinus* taught this, in a very positive manner, upon *general* grounds; but missed the *particular* proof of it which Sir C. Bell obtained. See Sir W. Hamilton's Historical Notices, in the Edinb. Phil. Journ., July, 1843.

exception taken by the whole mass of the German physiologists to Dr. Hall's doctrines; and we despair of their adhesion to them being ever gained, until they have been convinced of the *anatomical distinctness* of the excito-motor system of nerves; and this we do not think they will ever be by the study of the Vertebrata alone, because the two systems are there so blended together, as to render it impossible to isolate them for the purposes of experiment. In showing, therefore, that the sensori-volitional and excito-motor systems of nerves are structurally distinct in the Articulata, and that their respective functions can be separately experimented upon, we think it evident that Dr. Carpenter and Mr. Newport have done what Dr. M. Hall has *not* done; and what, when the results of their labours are duly considered, must gain assent to Dr. Hall's doctrines in many quarters where it is at present withheld. They have, in fact, done for the excito-motor and sensori-volitional systems, precisely that which Sir C. Bell did for the sensory and motor nerves; and just as Sir C. Bell would have found it difficult, perhaps impossible, to prove the structural distinctness of these in any other class than the Vertebrata, in which the trunks may be distinguished at their roots into afferent and efferent,—so is it difficult, perhaps impossible, to determine the distinctness of the sensori-volitional and excito-motor systems in any other class than the Articulata, in which the division of the nervous trunks at *their* roots, into the reflex and cerebral, can be made evident, both by anatomical and experimental investigation.

We hope that we shall not be thought to depreciate the merit of Mr. Grainger's labours in what we have now said; but we believe that his confidence in his own account of the anatomy of the spinal cord of Vertebrata, has been rather shaken by the recent observations of Dr. Stilling. To this subject we shall presently return, when giving an account of Mr. Newport's examination of the centipede.

The following is the principal novelty to be found in the sections of Dr. Hall's memoir devoted to the Anatomy and Physiology of the true Spinal Marrow. It relates to the nature of the œsophageal portion of the act of deglutition, on which opposite opinions have been expressed by Prof. J. Reid and Prof. Volkmann.

"This discrepancy I believe to originate in too exclusive views of the subject. Prof. J. Reid observed that after the division of the pneumogastric nerves above origin of the superior laryngeals in the rabbit, the principal part of the parsley eaten by the animal remained in the œsophagus, a few leaves was all that reached the stomach; [whence he inferred that the muscular contraction of the œsophagus is of a reflex character.] Prof. Volkmann concludes, from experiments made on the calf, in which the pneumogastric nerves were divided, and on the frog, in which the brain and spinal marrow were destroyed, that the deglutory movements of the œsophagus do not depend on the pneumogastric nerves. The fact is, I believe, judging from my own experiments, that the action of the œsophagus in deglutition is of a double or mixed character—prompt under the influence of the pneumogastric nerves, and of a slower character under the immediate influence of the peristaltic action of its muscular fibres. These views are supported by the following experiments:—The pneumogastric nerves were laid bare in a rabbit, and a considerable portion was removed; a little cabbage was then given. Deglutition seemed to be perfect at first; soon, however, uneasiness and a sort of cough was induced, a little of the cabbage with mucus being expelled. The rabbit was killed by a blow on the back of the neck. On examination the stomach was found replete with bran, &c., and the œsophagus with the green cabbage; not a particle of the latter had reached the stomach, but a little was found in the larynx and trachea. The peristaltic movements of the œsophagus were very marked, and expelled the cabbage through the lower extremity when this was cut. A *comparative* experiment was tried. Two rabbits were taken, and a portion of each pneumogastric nerve was removed in *one*. A very little cabbage was then given to both. In twenty minutes they were killed. In the rabbit in which the nerves were entire the cabbage was found entirely in the stomach, the œsophagus being quite empty. In the other a very little of the cabbage had passed into the stomach, whilst the œsophagus contained it from beginning to end. The action of the œsophagus under the influence of the pneumogastric nerves is, like that of pharynx, rapid and energetic. Its action after the nerves are divided is slow and peristaltic, like that of the intestine. The latter is obvious in the œsophagus separated entirely from the animal, and of sufficient power slowly to expel its contents." (pp. 53-5.)

We have little doubt that this is the true view of the matter; and that it is to be extended to the stomach also, with this difference, that in the latter the peristaltic movement is the prodominant action. It has been clearly proved that the pneumogastric *does* act upon the muscular coat of the stomach, since distinct contractions of that viscus have been repeatedly excited by irritation of the nervous trunk. But it also seems indubitable that the influence of the pneumogastric is not essential to the movements of the stomach; since Prof. J. Reid has ascertained that digestion and the passage of the digested aliment into the intestinal canal, which cannot be effected without these movements, may take place with almost their normal regularity after section of the pneumogastric on both sides. There is therefore a very beautiful gradation in the character of the movements concerned in the ingestion of aliment, as we trace them from the mouth downwards. Those by which it is introduced into the mouth are voluntary, in the higher animals at least; the act of pharyngeal deglutition is purely reflex; that of œsophageal deglutition is principally reflex, but partly peristaltic, or independent of nervous agency; the contractions of the stomach are chiefly peristaltic, but partly reflex; and those of the intestinal canal are still more restricted to the peristaltic character, but are probably in some slight degree reflex also.

In speaking of the connexion between the lower part of the spinal cord and the actions of the generative apparatus, Dr. Hall remarks:

"In connexion with these latter subjects I may observe that the next improvement in the obstetric art will, I believe, arise from the application of our knowledge of the excito-motor principle to that department of medicine. Remedies in the cases of sterility and of lingering labour, of atonic hemorrhagy, and other forms of inertia of the uterus will probably be found in some of the excitants of the excito-motor power." (p. 56.)

We rather wonder that Dr. Hall should not have adverted to the operation of several of the means employed by the accoucheur to bring about the contraction of the uterus in cases of hemorrhage after parturition, as examples of this kind of action. Every one knows the efficacy of friction upon the abdominal surface; where this fails, the sudden contact of the hand, previously cooled by immersion in cold water, will often produce the effect; if this should not succeed, the uterine contraction may often be brought about by the sudden immersion of the patient's hands in cold water; and the continuance of the hemorrhage requires the still more violent remedy of a dash of cold water over the surface of the trunk. Or the application of the child to the nipple has been said to produce uterine contraction, when other mild remedies have been unsuccessfully tried. It can scarcely be necessary to point out, that reflex action is evidently the channel of all these operations. The subject is afterwards slightly noticed in the section on the *therapeutics* of the true spinal system, which contains many valuable hints regarding the treatment of various diseases, particularly those of a convulsive nature. The importance of the *alternation* of the impressions of *heat* and *cold* in the treatment of congenital asphyxia, is particularly dwelt upon; and ample proof of its effects has been afforded by two papers by Mr. Simpson and Mr. Barlow, in the 'Lancet,' for 1842 and 1843. The necessity of *continuing* the resuscitating means for some time after respiration has been established, with the view of preventing a relapse into secondary asphyxia, is also strongly enforced:

"Perseverance is not less necessary in this case than in that of poisoning from opium. The blood is still poisoned; and a slight comparative failure in the respiration, as from sleep, may add to the dose of poison, (carbonic acid,) and prove fatal. My friend Mr. H. Smith has made a most important remark: *after* the partial establishment of respiration—therefore *after* inflation of the lungs—secondary asphyxia may prove fatal, and the life of a supposed criminal mother may be placed in fearful jeopardy, even by the medical evidence! Next to the remedies which have been noticed, the exposure of the face especially to a current of cold air will prove most important; and even after the infant is restored to animation, and clothed, its *face* should be freely exposed in a cool atmosphere. The fan may also prove of great assistance; the sudden gusts induced by it are especially useful. It is not in asphyxia only that these measures are important; in nausea and vomiting, in faintishness and syncope, in various sudden seizures, as in the convulsive diseases both of children and adults, they are equally useful. The cold breeze is the best remedy against sea-sickness, and extremely useful in asthma; the dashing of cold water on the face is the most efficacious remedy in syncope. In one case my friend Dr. Heming kept off the convulsive attack in an infant for a very long time by watching the premonitory symptoms, and sprinkling cold water on the face or surface. It will be remembered that Dr. Denman did the same thing in a case of puerperal convulsion. The larynx is opened, inspiration was excited, and the fit prevented." (pp. 65-6.)

In a subsequent section Dr. M. Hall again discusses at some length the nature of the act of respiration, with the view of setting right some misconceptions which have been entertained in regard to his view of it. As we have not partaken in these, however, we need not again take up the subject, but shall simply quote the following experiment, which shows how much the respiratory act may be excited by stimuli acting through the nerves of the general surface.

"If we remove the cerebrum and divide the pneumogastric nerves in a young kitten, the number of the acts of respiration may be reduced to four in a minute, in spite of the circulation of venous blood; but, by directing a stream of air on the animal, or irritating various parts of the general surface, we may excite twenty or thirty acts of respiration within that space of time." (p. 78.)

In a subsequent section "On the respiratory movements in asphyxia," Dr. Hall brings forward the novel doctrine, that whilst the ordinary movements of respiration are *reflex*, being excited through the medium of appropriate incident nerves, those which occur in asphyxia are of *centric* origin, being occasioned by the circulation of venous blood in the medulla oblongata. Without doubting that this last cause has a most important, agency, we must own that we cannot see the evidence of its *exclusive* operation; and there is a little more of dogmatism than of argument, in the manner in which this doctrine is propounded.

With the exception of an Appendix, containing a recapitulation of the positions previously advanced, the memoir contains nothing that has not now been placed before our readers in some form or other; and they will perhaps share with us in the feeling of surprise, that Dr. Hall should have thought it desirable to publish it in its present form. This, however, is his own affair; and though we may fairly say that we expected to have found more novelty in it, we have no right to complain that one who has done so much for physiology as Dr. M. Hall, should now be disposed to rest awhile, to give time for his labours to be appreciated, and aid, by the explanation of apparent difficulties and the correction of trifling errors, in promoting their general reception. This we take to be the purpose of the memoir, and we cordially hope that it may be successfully accomplished. We have a fellow-feeling with Dr. Hall in his concluding remarks:

"Every encouragement should be given to the diligent and devoted investigator; every obstacle, every kind of injustice, every source of disgust and of indignation, should, for the sake of science, for the honour of our institutions, be removed. The physician who devotes himself to investigation especially makes a thousand sacrifices. His path requires cheering, and should not—as it need not be—unjustly obstructed or beset by thorns." (p. 94.)

If *we* have erred in this respect towards Dr. M. Hall, we here, and once for all, offer him our sincerest apologies; that we have made the *amende honorable*, by doing all in our power to promote the reception of his views when once convinced of their truth, we think our readers will freely admit.

II. We now proceed to notice that part of Mr. Newport's memoir which bears on the main subject of Dr. Hall's. On some future occasion we hope to do more justice to Mr. Newport than we have hitherto done, by noticing more at length the various admirable anatomical and physiological memoirs with which he has enriched the 'Philosophical Transactions' during the last ten years. Although it may be anticipated, from what has been stated above, that Dr. Hall will probably regard Mr. Newport's researches on the nervous system of the Articulata as of no great value in relation to his peculiar views, we are disposed to consider them of very great importance indeed; and we think he cannot but feel gratified in having what so many others as well as ourselves will look upon as a most important confirmation or corroboration of his doctrines, worked out by an inquirer of such acknowledged accuracy, and whose claims to physiological discovery are only second to his own.

The evidence adduced by Mr. Newport, in the paper whose title is prefixed to this article, of the existence of a distinct set of nerves ministering to reflex action, appears to us of the most satisfactory character, and such as it is scarcely possible to withstand. It may be asserted, however, that this evidence only applies to the Articulated classes; and that it affords no support to the idea that such nerves exist in Vertebrated animals, in which they have not yet been demonstrated. But upon the same reasoning it might be asserted, that we have no ground to believe in the existence of distinct sensory and motor nerves in the Articulata, because their separate existence has only been proved in Vertebrata;—the fact being, that the four sets of nerves, of whose existence we feel well assured, are differently bound up together in these two groups of animals, as the following diagram will show:

| In VERTEBRATA. | *Distinct Classes of Fibres.* | In ARTICULATA. |
| --- | --- | --- |
| Afferent fibres, united in *posterior* roots of spinal nerves. | 1. Sensory<br>2. Volitional | Form continuous fibrous tract passing on to the *brain*. |
| Efferent fibres, united in *anterior* roots of spinal nerves. | 3. Excitor<br>4. Motor | Form distinct system of nerves, connected with *ventral* ganglia. |

In the *posterior* roots of the spinal nerves of Vertebrata both classes of *afferent* fibres,—those which are merely excitors of reflex action, and those which convey sensory impressions to the brain, are bound up together; and in their *anterior* roots both classes of *efferent* or *motor* fibres are bound up together,—those which excite the muscles to reflex action, and those which convey to them the determinations of the will. If we had no other anatomy of the nervous system to look to, we might never be able to prove satisfactorily that this double function exists in each root. But in the articulated animals we have a different arrangement; the excitor and motor nerves of the reflex system being anatomically distinguishable from those which minister to sensation and volition, although their sensory and excitor nerves cannot be distinguished as *afferent* fibres from the two classes of motor or *efferent*.

It is probably well known to many of our readers, that, in his former papers, Mr. Newport had described the nervous column of the articulata as consisting of two very distinct *tracts*; one of these containing white or fibrous structure only, and passing continuously *over* the ganglia; the other being studded with ganglia (each ganglion containing gray matter or that which is analogous to it,) at intervals. From both these tracts proceed fibres, which enter the roots of the nerves; every one of which has, therefore, a connexion with each division of the nervous cord,—the ganglionic and aganglionic. By Mr. Newport this double connexion was formerly viewed as analogous to the double connexion of the spinal nerves with the spinal cord of vertebrata; the roots that enter the ganglionic column (which is the nearest to the ventral* parietes of the animal) being regarded by him as analogous to the posterior roots of the spinal nerves, and therefore sensory; whilst those that are connected with the aganglionic column (which is the one nearest the visceral cavity) were considered as analogous to the anterior roots of the spinal nerves, and therefore motor.

On the other hand, Dr. Carpenter was led, by his comparison of the nervous system of the Articulata with that of the Mollusca, and by his desire to find a justification (if possible) for the doctrine of a distinct system of excito-motor nerves in the anatomy of the Invertebrata, to attribute very different functions to these parts respectively. Taking the anatomical statements of Mr. Newport as his chief guide, he regarded the white or fibrous tract as exclusively of *cerebral* origin, establishing the relation between the cephalic ganglia and the nerves of the body; and as he restricted to these ganglia alone the functions of sensation and

* It is now generally understood that the body of an articulated animal is altogether inverted; so that its so-called *ventral* surface is really its dorsum.

volition, he regarded this tract and the branches proceeding from it as the *sensori-volitional* system of nerves. On the other hand, supposing the nerves which enter the ganglionic tract to be exclusively connected with its ganglia, and finding in the actions of decapitated and divided articulata ample proof of the high development of reflex phenomena in that group, he regarded the ganglionic column, with the branches proceeding from it, to constitute the *excito-motor* system of nerves.

The views to which Mr. Newport has been led by his recent inquiries harmonize most completely *in principle* with those put forth by Dr. Carpenter, though differing from them in certain details. The points of agreement and difference will be discernible from the extracts we shall give from this valuable paper; but we may as well briefly state them *in limine.* Mr. Newport's more recent anatomical inquiries, which have been prosecuted with means of research superior to those formerly in his possession, have led him to the discovery that, in the ganglionic column itself, two very distinct orders of fibres exist, besides those commissural fibres which unites its two sides. Of these, one set appears to be as continuous between the cephalic ganglia and the roots of the nerves, as are the fibres of the aganglionic tract, and must therefore be regarded as belonging to the sensori-volitional system, not to the excito-motor. And he considers it not improbable—although he has not been able to substantiate his opinion by experiments,—that *these* are the *sensory* fibres; whilst those of the *aganglionic* column are *motor*. Hence he in some degree holds to his former opinion; and we doubt not that Dr. Carpenter would be ready to agree with him on this point: since the essential doctrine entertained by the latter was, that *all* the fibres that connect the nervous with the *cephalic* ganglia are sensori-volitional, whilst those which connect them with the *ventral* ganglia are purely reflex,—a doctrine which is not in the least degree invalidated by the discovery, that *some* of the fibres believed by Dr. C. to belong to the latter system must be transferred to the former. In the existence of a *distinct set* of fibres, connected *only* with the *ventral* ganglia, and ministering exclusively to reflex action, Mr. Newport is now as firm a believer as Dr. C.; and the exactness of his anatomical analyses of the ventral cord, and of the inferences to which he has been led by numerous well-devised experiments, seem to place the correctness of this view beyond all reasonable doubt.

One other point may be here best adverted to. Mr. Newport's researches lead to the belief that in no instances do the nervous fibres *terminate* in ganglionic matter by free extremities. He finds them, while *passing through* the mass of nucleated cells intermingled with a plexus of blood-vessels (of which this substance appears essentially to consist), undergoing considerable changes in their aspect; but after they have emerged, they recover their usual appearançe. So that it seems as if the afferent and efferent fibres were really continuous with each other, the change in their function, as well as in their direction, being effected during their passage through the gray matter: just as the arteries and veins are continuous, the change in the character of the fluid they convey, as well as in the direction of its movement, being effected during its transit through the capillaries. The inquiries of other observers have lately seemed to tend towards the same view; and it seems to explain much, that it would otherwise be difficult to reconcile with our notions of the distinct system of excito-motor nerves, in the researches of Dr. Stilling on the structure of the spinal cord, of which we give an account in another article.

As we are necessarily limited as to space, we shall not make any extract from Mr. Newport's account of the aganglionic column, which contains no novelty of importance; but shall proceed at once to his description of the ganglionic tract:

"The *inferior longitudinal,* or *ganglionic* set of fibres of the cord, affords many interesting considerations. It is placed, exactly as in insects, on the under surface, but like the upper series it is narrower than the whole cord, of which it forms a part. It is formed of a longitudinal series of fibres, like the upper tract, beneath which it is placed, and from which it is divided by some of the fibres that pass transversely through the cord, and which enter into the composition of the nerves from the ganglion on either side. It appears also to receive filaments from the upper series, and perhaps others are sent from it to the upper, thus decussating each other in the middle substance of the cord, where these two longitudinal series are in close apposition; since it is almost impossible, even in the large nervous cord of Scolopendra, to separate these two tracts from each other, although their distinctness is evinced in their relative size and longitudinal lines of separation. But there is one fact of great interest in regard to this ganglionic series of fibres. Almost the whole of the fibres of which it is composed are traceable, in the Iulidæ, directly through each enlargement of the cord, which they mainly assist to form. At the anterior part of each enlargement the diameter of each fibre, or fasciculus of fibres, appears to be slightly increased, and its structure becomes more softened and delicate. While passing through these ganglionic enlargements, occasioned chiefly by their own increased diameter, the fibres take a slightly curved direction outwards, and then inwards, but are reduced to their original size, and assume the longitudinal direction on again forming the aganglionic portion of this tract of the cord." (p. 249.)

The fibres described in this extract, as well as those of the aganglionic column, appear to belong to the sensori-volitional system. We now quote Mr. Newport's accounts of those which seem to minister exclusively to reflex action. These fibres, which were previously undescribed, form the sides of the cord, in the interspace between the ganglia, or between certain nerves distributed from them; and are termed by Mr. Newport *the fibres of reinforcement of the cord.* The great importance of this anatomical fact leads us to extract the description in full, although at the risk of exceeding our limits.

"*The fibres of reinforcement of the cord* form the lateral portions of the whole nervous cord of the body, and enter into the composition of all the nerves. They constitute, as it were, circles of nervous communication between two nerves that orginate from the cord at a greater or less distance; and form part of the cord in the interval between these nerves, and bear the same relation to the segments, individually, which the cord itself does to the whole body. They form part of the nervous trunks which come off from its upper, or aganglionic tract, as well as of those which proceed from the ganglionic enlargements in the lower, and in each instance they bound the posterior side of one nerve and the anterior of another, to which they proceed along the sides of the cord, forming in the interspace, a part of its structure. Each fibre may thus be traced from its peripheral distribution, in the structures of the external surface of the body, inwards, along the course of the nerves, on their posterior surface, to the cord, where its direction is altered from that of the nerve transversely inwards, to that of the cord on which it is reflected, and passes longitudinally backward; thus forming a part of its external surface until it arrives at the root of the nerve to which it is to be distributed, and along which it again passes transversely outwards, bounding the anterior side of the nerve to its distribution on the lateral surface of the body. These fibres of reinforcement form a large proportion of the whole cord, and enter into the composition of the upper, anterior, and part of the inferior surface of the root of every nerve, in their course inwards to the cord; and of its posterior and inferior surface on their again proceeding outwards. In this manner these fibres of reinforcement connect all the nerves of the cord on one side of the body, as the corresponding fibres do those on the opposite side. They form, as it were, double, treble, or quadruple circles, one within the other. Thus the fibres that pass inwards along one nerve may proceed along the cord to pass outwards again on the front of a second, a third, or a fourth, thus linking the segments in one continued series of nervous communications, independent of the brain. But these communications exist only between nerves on the same side of the body, and not between those on the opposite. The *commissural* nerves connect the opposite sides of each individual segment, as those of *reinforcement* do the same sides of two separate segments.

"Every nerve from a ganglionic enlargement of the cord is thus composed of *four sets* of fibres, an upper and an under one, which communicate with the cephalic ganglia; a transverse or *commissural,* that communicate only with corresponding nerves on the opposite side of the body; and a lateral set that communicate only with nerves from a ganglionic enlargement on the same side of the body, and form part of the cord in the interspace between the roots and the nerves. It is by the successive addition of these lateral portions of the cord, that its size is maintained almost uniformly throughout its whole length in the elongated bodies of the Myriapoda. On examining the cord very closely, I have reason to believe that the upper and inferior sets of longitudinal fibres, the ganglionic and the aganglionic, are somewhat smaller at their posterior than at their anterior extremity, a circumstance readily understood in the fact that successive series of filaments are given off from them at each distribution of nerves from the ganglonic enlargements; while the relative size of the lateral portions of the cord appears to be greater in the posterior than in the anterior. On this account I have named these lateral fibres *fibres of reinforcement of the cord.*

"In regard to the identification of these fibres, it may be well further to state, that their separate existence is indicated chiefly at the postero-lateral margin of the ganglia where they are seen to form part of the nerves and cord without passing upwards to the brain. In other parts of their course they are not distinguishable by colour, and very rarely by any longitudinal line of separation, from the fibres which form the inferior longitudinal series, or portion of the cord, to which they are approximated; but from which they are believed to be distinct, from the fact, that they do not ascend with them to the brain. Their function must be regarded only as *reflex;* entirely independent of sensation, but capable of being excited into action by external causes. The existence of these lateral fibres in the cord may now fully explain the reflected movement of parts anterior or posterior to an irritated limb on the same side of the body, as the commissural ones do the movement of parts on the side opposite to that which is irritated." (pp. 250-2.)

In order to demonstrate the respective functions of these systems, a number of well-devised experiments were performed by Mr. Newport; the results of which we cannot but regard as most satisfactory. We shall quote the *fifth* as one of the most striking and conclusive:

"*Experiment* 5. The cord alone was divided in the *fourteenth* and also the *twentieth* segment, and the intervening portion was destroyed by breaking it down with a needle. The animal exhibited in the anterior part of its body all the evidences of perfect volition. It moved actively along, turning itself back on either side repeatedly, as if to examine the anterior wounded portion, which it felt again and again with its antennæ, and when attempting to escape, frequently turned back as if in pain and aware of some hindrance to its movements, but it seemed perfectly unconscious of the existence of the posterior part of its body, behind the first incision. In those segments in which the cord was destroyed, the legs were motionless, while those of the posterior division, behind the second incision, were in constant, but involuntary motion, the movements being similar to those of walking or running, uniformly continued, but without any consentaneous action with those of the anterior part, by which locomotion was performed, dragging the posterior divisions of the body after them. When the animal was held by the posterior segments, reflex actions were excited in the legs, and powerful contractions and gyrations of the whole animal were performed in those segments; but these movements appeared to be entirely the result of reflex actions of the muscles, since exactly similar ones took place in the whole body in decapitated specimens. At the expiration of *twelve* hours the most perfectly voluntary acts were performed by the head and anterior division of the body, such as locomotion forwards or to either side, avoidance of any obstacle, touching it with the antennæ, which were in rapid action as in an uninjured animal, and attempting to reach and to climb up an object presented to it, but not in immediate contact with it. But reflex movements alone existed in the posterior division, in which the legs were very slowly moved, even when the animal was not progressing. Brisk actions were now more easily excited in them than at first, either by contact with the segments, by irritation of one or two of the legs themselves, or by a sudden current of air. By these means, when the animal was lying still, actions were immediately excited in all the legs of the posterior part of the body anterior and posterior to those which were irritated, and these actions were induced in those of both sides of the body, but appeared to commence on the opposite side, in the legs corresponding to those which were first irritated. In *eighteen* hours the anterior part of the body was quite dead, no motions whatever could be excited on it, either voluntary or reflex; but reflex actions were then readily excited in the posterior, and also slightly so by mechanical irritation, even at *twenty-four* hours." (pp. 267-8.)

This experiment, and most of the others, were made upon the *Iulus*, one of the vermiform myriapoda, in which the cephalic ganglia are small in proportion to the rest, and the excito-motor system is evidently the predominant one. But they were repeated, with corresponding results, upon one of the higher species, the *lithobius forficatus*, in which, from the peculiar structure of the head, the cerebral ganglia could be removed from the body, without removing the medulla oblongata (or penduncle of the ganglia) from which the nerves are given to the parts of the mouth. The following are Mr. Newport's general conclusions from the whole:

"These experiments seem to lead to the conclusion that the seat of volition is solely in the supra-œsophageal ganglia or brain of these animals, since all direction of purpose, all avoidance of danger, all control over the movements of the body, either of speed or change of direction, are lost when these are much injured or removed. Volition ceases quickly when they are severely wounded, and is greatly diminished even when one only is slightly affected. This latter fact is indicated by the loss or diminution of purpose, and by the gyratory movements of the body. The experiments seem also to show that sensation may remain after the injury or removal of one lobe of the brain, as was proved by the retraction of the antenna when slightly touched on the uninjured side of the head, and by the cleansing and excited act of drawing it constantly through the mandibles; and further, that pain is felt when the cerebral lobes are injured, as when the needle was applied to them after the antennæ had been removed. They lead also to the conclusion, that all the phenomena which occur in the posterior parts of the body after the brain and cord have been separated are reflex or excited, and that these are most intense at the two extremities of the cord—the medulla oblongata, and the terminal ganglion; and further, that the reflex phenomena are always excited and do not occur spontaneously, and that their intensity is greater in proportion to the stimulus applied, and gradually diminishes until they entirely cease, or are re-excited, precisely as already shown by Dr. Hall in the Vertebrata.

The experiments both on the Iulus and Lithobius seem further to show, that the reflected movements cease first in the anterior part of the cord and its ganglia, and that they are retained longest in the posterior; that the movements are most powerful and continue longest when the cord is entire, the brain alone being separated from it; and that they entirely cease sooner in proportion to the greater number of parts into which the cord is separated: further also, that the reflex phenomena are less readily excited in the anterior part of the cord, while it is still in connexion with the brain, and that they cease entirely soon after the cessation of volition in that organ; as in those experiments in which only a very short portion of cord was removed with it from the body.

"Many of the phenomena are precisely similar to those which have heretofore been observed in the Crustacea. They agree in the circumstance that violent contractions of the segments and limbs, both anterior and posterior to a ganglion, are induced by irritation of that ganglion, both when connected with the brain and when insulated from it, thus proving these movements, in the latter instance, to be reflex; but there is as yet no direct *proof* that sensation does not also exist in these ganglia.

"The general results of these experiments tend to confirm the belief that the fibres now pointed out in the composition of the cord and ganglia, and which cannot be traced to the brain, are those by which these movements are executed independently of that organ; and further also, that the reflex phenomena are most intense, most easily induced, and are of longest duration, in those animals of low organization, in which the volume of brain bears the smallest proportion to that of the whole nervous system, in which also volition and sensation are of small amount, and which have the body formed of the greatest number of similar uniform parts or segments." (pp. 271-2.)

An important addition is made in a note.

"While this paper has been passing through the press, I have repeated these experiments, on the functions of the brain and cord, with still more conclusive results on the Cleoptera, Orthoptera, Hymenoptera, Neuroptera, Diptera, and other hexapod insects. The cord was divided between the first and second pairs of legs. The two posterior pairs of legs were immediately deprived of volition, and exhibited only reflex actions, while the anterior pair gave marked indications of being as completely under the influence of volition and sensation as in the uninjured animal. The cord was then divided between the first pair of legs and medulla oblongata, when these legs also were deprived of volition, and exhibited only reflex actions like the posterior.

"Other experiments made on the brain itself, by removing that organ, or by simply separating it from the medulla oblongata and cord, without decapitating the insect, fully confirmed the experiments on the Myriapoda, in proving that the supra-œsophageal ganglia have the functions of a true brain, and are the sole seat of sensation and volition; and that although, when this organ is removed or is insulated from the cord, a regular, combined, and consentaneous series of muscular actions can be excited in the limbs, and locomotion induced, these acts are then entirely automatic, and are performed without the intervention of sensation or volition." (p. 272.)

We shall only add the following passage, extracted from Mr. Newport's general summary, which directs attention to a very important subject that is now attracting much attention,—the connexion of the *nutrition* and *vital actions* of a part. This subject was noticed by Mr. Newport in a former part of the memoir, when describing the cord and changes of the nervous system in *polydesmus* and *geophilus*. We, ourselves, have long entertained the same notion, believing that the ganglionic matter, if the centre of the *actions* of the nervous system, must be the centre of its *nutrition* also. But this ganglionic matter must be looked for, not only in the *centres* of the nervous system, but in its *periphery*, the spots in which the afferent fibres originate.

"The ganglia of the cord are regarded not only as analogous, anatomically, to the enlarged portions of the cord in Vertebrata, but, physiologically, as centres of reflexion, agreeably to the views of Dr. Carpenter; and they also possess a still more important character and function in the nervous system, that of being the centres of growth and nutrition to the cord and nerves, the nuclei contained in them being perhaps the sources of supply and nourishment. This is shown from the fact that, in these parts, the fibres of the cord are softer and larger than in the rest of their course; and are elongated during the growth of the body, and the development of new segments; as is seen in the *Polydesmidæ* and *Geophilidæ*, families from the two divisions of Myriapoda. These additional facts fully accord with the already ascertained mode of development *by extension*, or simple growth of the segments." (p. 300.)

---

## Figures and Tables

**Figure f1:**